# Preparation of g-C_3_N_4_/TCNQ Composite and Photocatalytic Degradation of Pefloxacin

**DOI:** 10.3390/mi14050941

**Published:** 2023-04-26

**Authors:** Qiuping Li, Nuan Wen, Wu Zhang, Liansheng Yu, Jinghui Shen, Shuxian Li, Yuguang Lv

**Affiliations:** College of Pharmacy, Jiamusi University, Jiamusi 154007, China

**Keywords:** TCNQ, graphite phase carbon nitride, pefloxacin, photocatalysis

## Abstract

g-C_3_N_4_ and g-C_3_N_4_/TCNQ composites with different doping levels were prepared using the copolymerization thermal method with melamine as a precursor. XRD, FT-IR, SEM, TEM, DRS, PL, and I-T characterized them. The composites were successfully prepared in this study. The photocatalytic degradation of pefloxacin (PEF), enrofloxacin (ciprofloxacin), and ciprofloxacin (ciprofloxacin) under visible light (λ > 550 nm) showed that the composite material had the best degradation effect on PEF. When TCNQ doping is 20 mg and catalyst dosage is 50 mg, the catalytic effect is the best, and the degradation rate reaches 91.6%, k = 0.0111 min^−1^, which is four times that of g-C_3_N_4_. Repeated experiments found that the cyclic stability of the g-C_3_N_4_/TCNQ composite was good. The XRD images were almost unchanged after five reactions. The radical capture experiments revealed that ·O^2−^ was the main active species in the g-C_3_N_4_/TCNQ catalytic system, and h^+^ also played a role in PEF degradation. And the possible mechanism for PEF degradation was speculated.

## 1. Introduction

Antibiotics are a class of secondary metabolites produced by microorganisms or higher animals and plants in life with anti-pathogen or other activities [1,2]. Antibiotics enter the ecological environment because of their high-water solubility, lack of degradability, and other characteristics; they accumulate in the environment, and seriously destroy the balance of the ecological system [3,4]. Pharmaceutical wastewater in China has a large output and a wide distribution range every year, among which antibiotic wastewater accounts for the central part. Common antibiotics include quinolones, tetracycline, etc. [5]. Quinolone antibiotics are widely used, and their migration and transformation in the environment have received significant attention. According to the differences in antibacterial properties, the third-generation drugs represented by Pefloxacin (PEF), Norfloxacin (NOF), ciprofloxacin (CIP), and Levofloxacin (LEVX) are the most commonly used [6,7]. Quinolones (4-Quinolones), also known as pyridyl or pyridyl acids, are synthetic antibacterial drugs containing the basic structure of 4-quinolone. As broad-spectrum antibacterial drugs, quinolone antibiotics are used to treat infectious diseases in humans and animals due to their strong sterilization ability, good absorption by the body, and common adverse reactions. In recent years, the abuse of antibiotics has been severe [8,9,10]. Such drugs cannot be wholly metabolized after ingestion by organisms, and widely exist in pharmaceutical industry wastewater, medical wastewater, animal husbandry wastewater [11,12], and even surface water.

The semiconductor material g-C_3_N_4_ has excellent properties, such as high chemical and thermal stability, a flexible electronic structure, and a moderate band gap (2.7 eV). Due to its good biocompatibility and other characteristics, g-C_3_N_4_ has attracted significant attention in the industry and is widely used in photocatalysis [13,14], electrochemical sensing [15], bioimaging [16], drug delivery [17], and environmental monitoring, etc. [18,19,20]. Briefly speaking, photocatalytic technology refers to converting light energy into chemical energy by semiconductor materials under ultraviolet and visible light radiation, and the quick degradation or mineralization of pollutants. Organic pollutants harm the natural environment and can rapidly decompose into tiny, harmless molecular substances. The photocatalytic efficiency of pure g-C_3_N_4_ is often limited due to its high exciton binding energy, insufficient solar absorption, low surface area, and the tendency of photogenerated electron/hole pairs to rapidly complex [21,22]. Therefore, the modification of g-C_3_N_4_ is significant.

Wu et al. [23] showed that carbon dot-modified hollow porous g-C_3_N_4_ nanospheres have excellent photocatalytic degradation of naproxen under natural sunlight irradiation due to the excellent upconversion properties of carbon dots that can absorb and convert low-energy photons. Xiao et al. [24] developed a simple and cost-effective strategy to anchor copper single atoms to tubular g-C_3_N_4_ by inserting sodium chlorophyll copper into supramolecular precursors and subjecting them to heat treatment. The Cu atoms can be coordinated with three in-plane N atoms or four N atoms residing between two adjacent g-C_3_N_4_ layers to establish Cu-Nx charge transport channels, thus significantly facilitating the transfer of photogenerated carriers between the in-plane and interlayer. Ding et al. [25] showed that the rate of photocatalytic degradation of 2-chlorodibenzo-p-dioxin by g-C_3_N_4_ modified with palladium nanoparticles was 3.7 times higher than that of g-C_3_N_4_. This is because the Schottky barrier appears between Pd nanoparticles and g-C_3_N_4_, which expands the light absorption of g-C_3_N_4_ and hinders the complexation of photogenerated electron-hole pairs.

Research has shown that 7,7,8,8-tetracyano-p-phenyldiquinone methane (TCNQ) is an excellent organic electron acceptor, and it can form relatively stable charge transfer complexes with many transition metals, alkali metals, and other materials with electron donors [26]. These metal–organic complexes have good electrical, magnetic, and optical properties [27,28]. There has been much research on the compounds involved in the formation of TCNQ. One is the synthesis and characteristics of “organometallic” charge transfer salts formed by TCNQ and other organic compounds. Another is the synthesis and properties of organometallic compounds formed by TCNQ and metal ions [29,30].

Xie’s group [31] successfully synthesized AgI @ TCNQ composites for the photocatalytic degradation of methylene blue (MB) and found that the degradation rate of the composites was 1.8 times higher than that of AgI.

This paper synthesized a g-C_3_N_4_/TCNQ composite, studied its photocatalytic performance, and applied it to the degradation of quinolone antibiotic PEF. This paper discusses the catalytic effect of different doping amounts and catalyst amounts and expounds on the active species, potential degradation mechanism, and cycle stability of g-C_3_N_4_/TCNQ composite samples. It provides an advanced and green method for degrading antibiotics in wastewater, which is of great significance for improving the environment polluted by antibiotic wastewater.

## 2. Materials and Methods

### 2.1. Reagents and Instruments

The 7,7,8,8-Tetracyano-p-benzoquinone methane (TCNQ) was purchased from Aladdin Co., LTD. (Beijing, China), the melamine was purchased from Aladdin Industries (Beijing, China), the Pefloxacin (PEF) was purchased from Shanghai Jining Industrial Co., LTD. (Shanghai, China) the anhydrous ethanol was purchased from Tianjin Kaitong Chemical Reagent Co., LTD. (Tianjin, China), and the acetone was purchased from Tianjin Kaitong Chemical Reagent Co., LTD. (Tianjin, China).

Muffle Furnace (KSY-6D-16), Shenyang Energy-saving Electric Furnace Factory; Ultraviolet Spectrophotometer (UV-2550), Shimadzu Company, Guangzhou, China; X-ray diffractometer (3DMAX-IIIC), Nippon Shinosu Co., LTD., Kyoto, Japan; Fourier Transform Infrared Spectrometer (NEXUS-670), Thermo Fisher Scientific, New York, NY, USA.

### 2.2. Synthesis of g-C_3_N_4_

Synthesis of g-C_3_N_4_: The preparation of g-C_3_N_4_ was carried out by a thermal condensation method using melamine as a precursor. After being thoroughly ground down, 4 g melamine was put into the crucible, with a cover loosely on it. Then it was placed in a Muffle furnace. Under atmospheric conditions, the heating rate was controlled at 20 °C/min, and the temperature was heated to 550 °C for 4 h. When the temperature cooled naturally to room temperature, it was removed from the Muffle furnace to obtain the light-yellow solid g-C_3_N_4_, which was ground into a powder for later use.

### 2.3. Preparation of g-C_3_N_4_ Copolymerized by TCNQ

Preparation of TCNQ doped g-C_3_N_4_: 4 g melamine and a certain amount of TCNQ were mixed up and evenly ground down, and then collected in the crucible, with a cover loosely on it. Then it was placed in a Muffle furnace. Under atmospheric conditions, the heating rate was controlled at 20 °C/min. It heated the temperature to 550 °C for four h. After cooling to room temperature, the obtained g-C_3_N_4_/TCNQ-X samples were taken out of the Muffle furnace and ground into a powder for later use, where x represented the added amount of TCNQ (x = 10 mg, 20 mg, 30 mg).

### 2.4. Photocatalytic Degradation of Pefloxacin in g-C_3_N_4_/TCNQ Composites

A 50 mg g-C_3_N_4_/TCNQ complex with different doping ratios was accurately weighed and dispersed in a 50 mL 10 mg/L PEF solution. The samples were dispersed under ultrasonic waves for 30 min to achieve the dispersion uniform and were placed in a dark environment for 30 min and magnetic stirring was used to achieve an adsorption–desorption equilibrium. Then, the xenon lamp (300 W) was set up to simulate the photocatalytic degradation experiment. Every 30 min, about 5 mL of the reaction liquid was taken for centrifuging twice. The absorbance of the supernatant was measured at 276 nm using an ultraviolet-visible spectrophotometer. The water circulation temperature had to be kept at about 20 °C to avoid the influence of temperature on the experiment.

## 3. Results and Discussion

### 3.1. XRD Analysis

**Figure 1:**
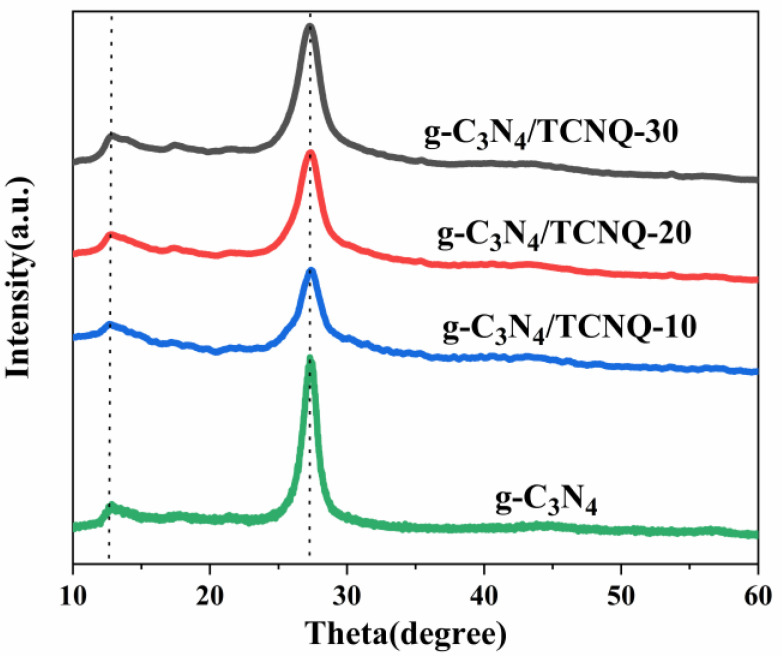
XRD patterns of g-C_3_N_4_ and g-C_3_N_4_/TCNQ.

An X-ray diffractometer can be used to determine the crystal structure diagram. Figure 1 shows the XRD patterns of the g-C_3_N_4_ and g-C_3_N_4_/TCNQ samples. It can be seen from the figure that distinct diffraction peaks appear at 13.1° and 27.4°, where the peak at 27.4° is the (002) peak of g-C_3_N_4_, reflecting the interlayer stacking structure of the aromatic triazine ring with an interlayer distance of d = 0.326 nm; the peak at 13.1° corresponds to the (100) peak of g-C_3_N_4_ s (100) peak, reflecting the in-plane structure of the triazine unit with an in-plane distance of d = 0.676 nm. The characteristic peak of the g-C_3_N_4_ material standard card (JCPDS-87-1526) is consistent [32]. The characteristic peaks of g-C_3_N_4_ did not shift significantly with the doping of TCNQ, which means that the g-C_3_N_4_/TCNQ samples can maintain the graphene-like in-plane and interlayer characteristics of g-C_3_N_4_, indicating that the graphene-like structure of g-C_3_N_4_ was prepared by simple thermal copolymerization.

### 3.2. FT-IR Analysis

**Figure 2:**
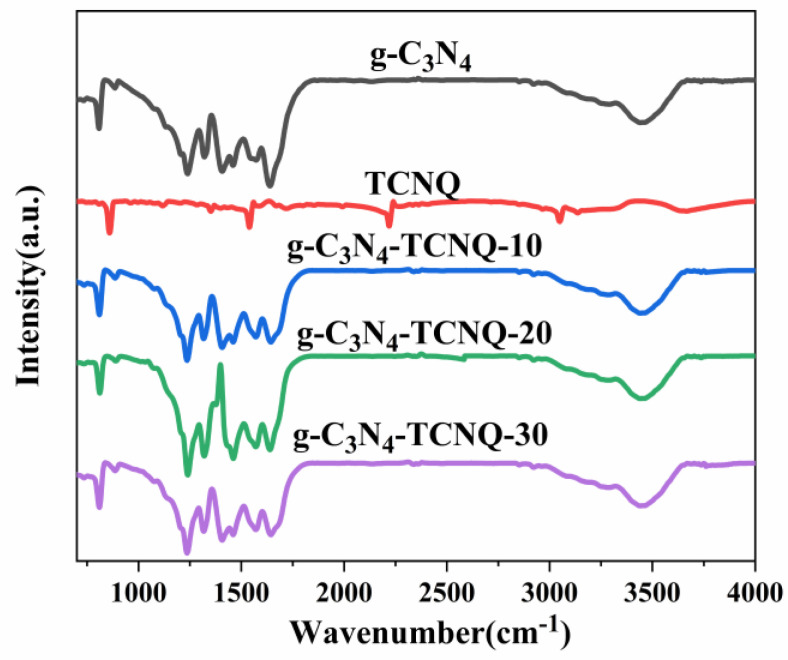
FT−IR images of g-C_3_N_4_ and gC_3_N_4_/TCNQ samples.

Infrared spectroscopy is used to analyze the functional groups of the sample and further possible chemical structures. Figure 2 shows the FT-IR images of g-C_3_N_4_ and g-C_3_N_4_/TCNQ samples. It can be seen from the figure that the characteristic spectra of all samples are similar. The absorption band located in the 1200–1700 cm^−1^ region is the characteristic resonance peak of the aromatic C-N heterocyclic repeating unit. In this range, peaks at 1240, 1318, and 1573 cm^−1^ correspond to C-NH-C, C-N and C=N keys in g-C_3_N_4_, respectively [33,34]. The absorption peak at 810 cm^−1^ corresponds to the structure of the 3-s-triazine ring (C_3_N_3_) [35]. In addition, the peak in the range of 3000–3500 cm^−1^ is an N-H bond [36]. The FT-IR results showed that TCNQ copolymerization did not change the chemical structure of g-C_3_N_4_. Because the doping amount is too low, no other response can be detected in g-C_3_N_4_ after TCNQ doping.

### 3.3. SEM Analysis

**Figure 3:**
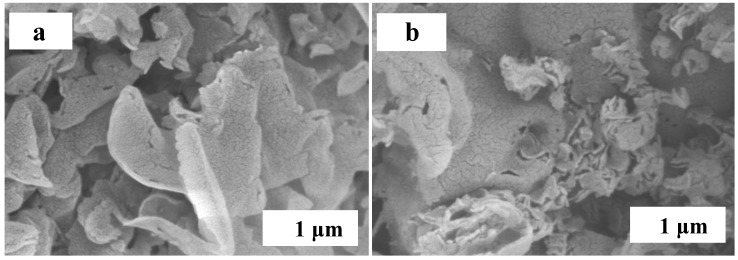
SEM images of (**a**) g-C_3_N_4_ and (**b**) g-C_3_N_4_/TCNQ-20.

A scanning electron microscope (SEM) was used to observe the surface morphology of the samples. Figure 3 shows the SEM of the g-C_3_N_4_ and g-C_3_N_4_/TCNQ-20 samples. The figure shows that g-C_3_N_4_ has a large size sheet structure, and the size and thickness of g-C_3_N_4_/TCNQ flake structures decreased, indicating that the copolymerization of TCNQ can significantly change the surface morphology of g-C_3_N_4_.

### 3.4. TEM Analysis

**Figure 4:**
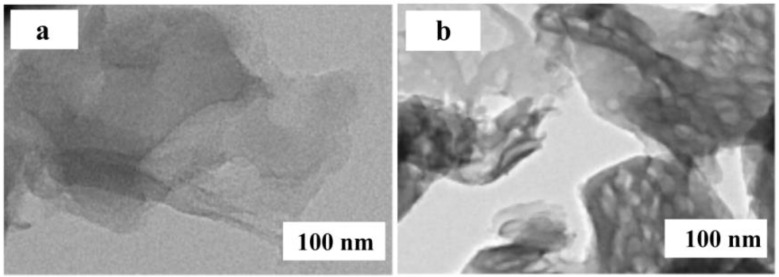
TEM images of (**a**) g-C_3_N_4_ and (**b**) g-C_3_N_4_/TCNQ-20.

Transmission electron microscopy (TEM) is used to observe the samples’ further morphology and structural characteristics. Figure 4 shows the TEM images of g-C_3_N_4_ and g-C_3_N_4_/TCNQ-20, from which it can be observed that the samples are all two-dimensional nanomaterials. The original g-C_3_N_4_ two-dimensional sheet nanosheet has a large size, smooth surface, and integrity. With the addition of TCNQ doping, the g-C_3_N_4_ nanosheet size reduces and more pore structures emerge on the surface, indicating that the process of TCNQ copolymerization can lead to the fragmentation of nanosheets and thus produce pores on the surface of g-C_3_N_4_. This result is consistent with the results of the XRD analysis.

### 3.5. DRS Analysis

**Figure 5:**
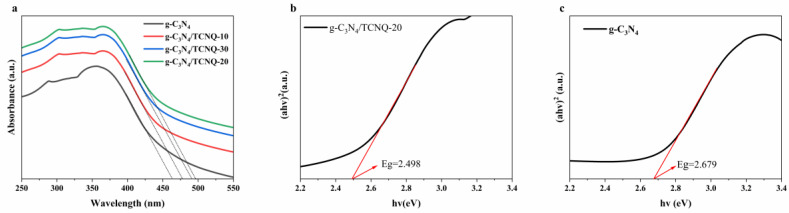
(**a**) UV-vis diffuse reflectance spectra, (**b**) forbidden band widths of g-C_3_N_4_/TCNQ composite samples and (**c**) forbidden band widths of g-C_3_N_4_ sample.

UV-vis diffuse reflectance spectroscopy (DRS) was used to study the light absorption of prepared samples. Figure 5 shows the DRS diagram of the g-C_3_N_4_ and g-C_3_N_4_/TCNQ samples. It can be seen from the figure that the absorption threshold of g-C_3_N_4_ samples occurs at 462 nm, and the corresponding bandgap width is 2.68 eV, which is consistent with the reported bandgap width of 2.7 eV [37]. The absorption thresholds of g-C_3_N_4_/TCNQ-10, g-C_3_N_4_/TCNQ-20, and g-C_3_N_4_/TCNQ-30 are 478 nm, 497 nm, and 491 nm, respectively, and the corresponding bandgap widths are 2.59 eV, 2.49 eV, and 2.52 eV, respectively. The corresponding band gap width also decreases. The absorption threshold of the sample is red-shifted, which may be caused by the nitrogen defect. The absence of an amino group will generate excess electrons in g-C_3_N_4_, making the carbon initially connected to g-C_3_N_4_ become C^3+^ and enter the conduction band, thus reducing the band gap width [38]. This indicates that TCNQ copolymerization can effectively narrow the band gap, increase the absorption of the visible light region, and increase the g-C_3_N_4_/TCNQ samples’ photoexcitation effectiveness and their photocatalytic activity, to a certain extent.

### 3.6. PL Analysis

**Figure 6:**
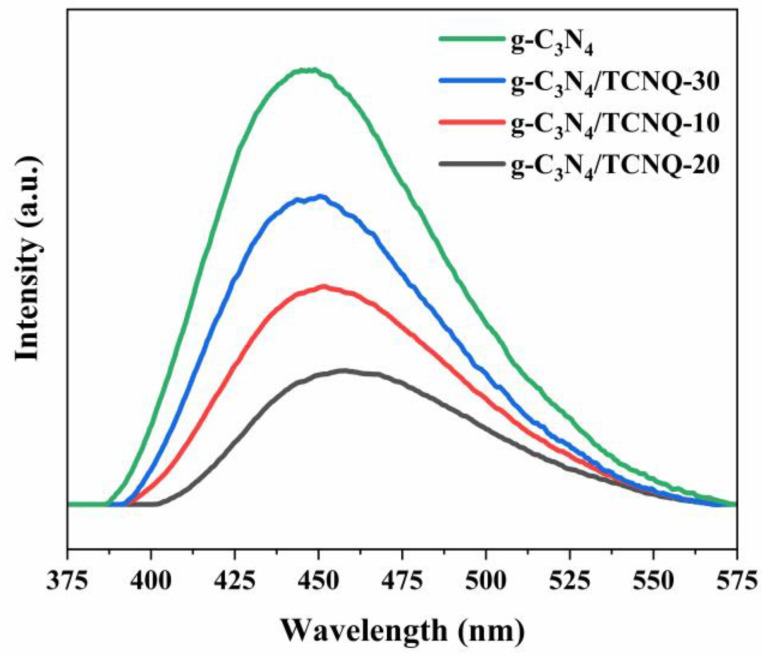
PL plots of the g-C_3_N_4_/TCNQ composite samples.

The recombination of photogenerated electron–hole pairs generates fluorescence (PL) spectra, so measuring the intensity of PL spectra can help us better understand the photogenerated carriers’ transmission and the recombination process in semiconductors. Figure 6 shows the photoluminescence spectra of g-C_3_N_4_ and g-C_3_N_4_/TCNQ samples. It can be seen from the figure that in the visible light wave region, all samples can generate fluorescent signals, indicating that electron–hole pairs can be generated when the material absorbs photons. This electron–hole pair is an essential step in inducing photoluminescence. With the increase of TCNQ doping, the intensity of the luminescence signal decreases gradually, and a photogenerated charge carrier recombination is prevented, which indicates that TCNQ doping can reduce the recombination rate of a photogenerated electron–hole and improve the separation efficiency. In general, the intensity of the fluorescence of the sample reflects the probability of a recombination of the generated electron–hole pair during the occurrence of a photocatalytic reaction. The higher the separation efficiency of the photogenerated electron–hole pair, the lower the recombination rate of the corresponding photogenerated charge carrier, the more conducive it is to the reaction of the electron–hole with the organic compound, and the higher the activity of the photocatalyst may be [39,40]. Moreover, when the doping amount of TCNQ increases, the PL peak position also shifts, consistent with the narrowness of the energy gap and the absorption redshift in the DRS spectrum.

### 3.7. Photocurrent Test

**Figure 7:**
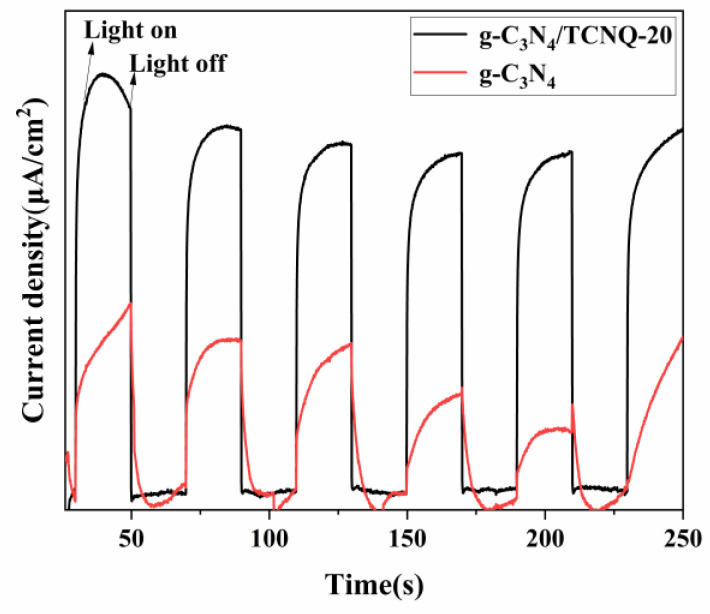
Transient photocurrent responses of g-C_3_N_4_ and g-C_3_N_4_/TCNQ composites.

The photocatalyst’s instantaneous photocurrent was measured using a standard three-electrode system with a prepared catalytic film as the working electrode, a platinum plate as the reactive electrode, mercuric chloride as the reference electrode, and 0.1 M sodium sulfate as the electrolyte. The photoelectric properties of the prepared photocatalytic materials were obtained by measuring the photocurrent under visible light. The transient photocurrent responses (I-T) g-C_3_N_4_ and g-C_3_N_4_/TCNQ prepared composite samples were measured. Figure 7 shows the I-T diagram of the two samples, from which it is apparent that when the light source is turned on, the current of both samples reaches the peak. When the light source is turned off, the photogenerated current decreases rapidly, indicating that the photogenerated electron holes in the material cause the current generation. The photogenerated current intensity of g-C_3_N_4_/TCNQ is much stronger than that of g-C_3_N_4_, indicating that TCNQ copolymer doping is beneficial to separate photogenerated electrons and holes, and can generate more charge carriers, which is conducive to the improvement of photocatalytic activity [41].

### 3.8. UV Absorption Spectroscopy

**Figure 8:**
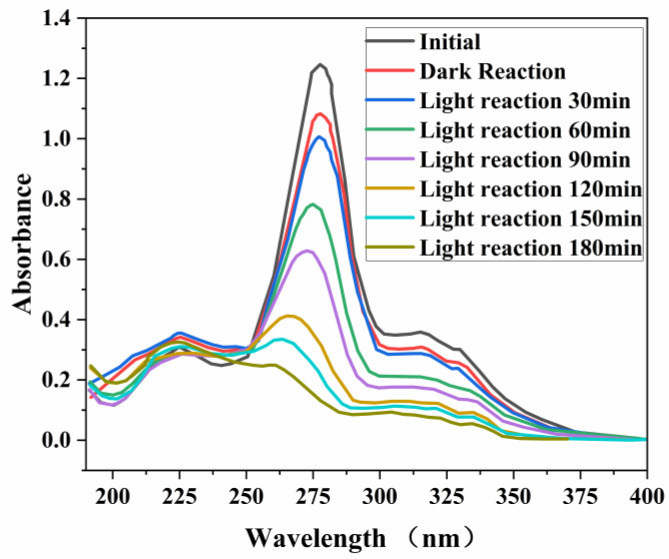
UV absorption spectra of PEF degradation by g-C_3_N_4_/TCNQ composites.

UV absorption spectra of PEF degradation by g-C_3_N_4_/TCNQ composites showed that (Figure 8) the positions of the absorption peaks of the initial sample and the full-wavelength scan of the sample in the dark reaction stage remained the same. Only the intensity was slightly reduced, indicating that the adsorption process does not change the original state of the substance but only absorbs the pollutant molecules to the surface of the photocatalyst. After the adsorption equilibrium, visible light was added. The position of the original strong absorption peak changed with the increase in time. The peak intensity gradually decreased, and the peak intensity still decreased in the case of almost no adsorption. This indicates that the photocatalytic process destroyed the structure of antibiotics and generated other substances. The absorption of PEF at 272 nm decreased with the increase in light time, and it can also be seen that the maximum wavelength of absorption increased with the increase in light time The absorption of PEF at 272 nm decreased with increasing light time, and it was also observed that the maximum wavelength of absorption decreased with increasing light time and showed a significant blue shift.

### 3.9. Influence of Different Drugs on Catalytic Effect

**Figure 9:**
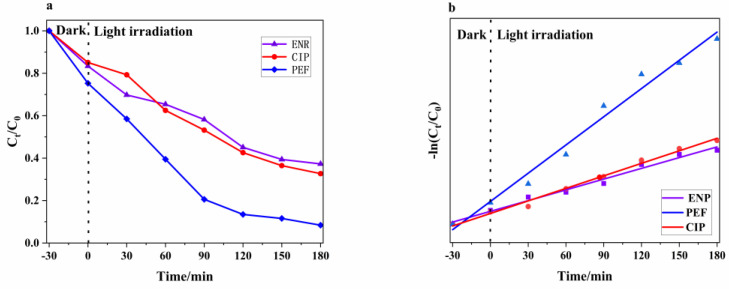
(**a**) Catalytic degradation of the different drug pairs by the g−C_3_N_4_/TCNQ composite (**b**) The kinetic curves of the first-order reaction for the different photocatalysts.

Figure 9a shows that: the g-C_3_N_4_/TCNQ-20 complex was taken and dispersed in PEF, ENR, and CIP drug solutions, and the samples were dispersed under ultrasonic waves for 30 min, and then dispersed uniformly in a dark environment with magnetic stirring for 30 min, called the dark reaction, and the dark reaction was finished and the light reaction was set to 0 points at the beginning.

After 180 min of photocatalytic reaction, D_PEF_ = 91.6%, D_ENR_ = 62.7% and D_CIP_ = 67.3%. The experimental results showed that compared with ENR and CIP, the sample has the highest degradation rate of PEF and the best catalytic activity. This paper discusses the photocatalytic degradation kinetics of PEF, ENR, and CIP and adopts the first-order kinetic method to fit them [42]. The expression is: ln(C_0_/C_t_) = kt, where C_0_ and C_t_ are the concentrations of PEF, ENR, and CIP at time 0 and t, and k is the first-order velocity constant. Figure 9b shows an excellent linear relationship between ln(C_0_/C_t_) and the photocatalytic reaction time t for each drug.

### 3.10. Influence of Different Copolymer Doping Amounts on the Catalytic Effect of PEF

**Figure 10:**
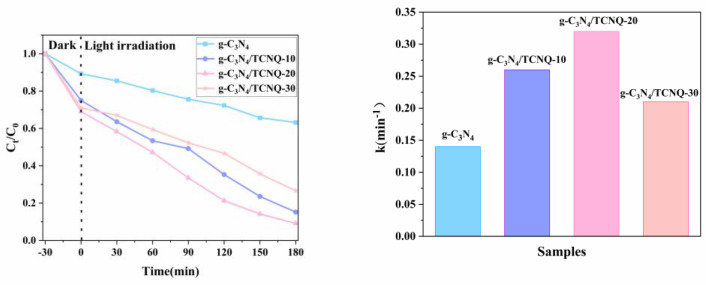
Photocatalytic effect of g−C_3_N_4/_TCNQ samples with different TCNQ doping ratios.

As can be seen from Figure 10, the dark reaction was performed for 30 min first, and g-C_3_N_4_ had a deficient photocatalytic activity with a D_PEF_ = 36.8% as the time increased. While g-C_3_N_4_ can effectively absorb visible light, the e^−^-h^+^ pair’s recombination rate is very high, inhibiting its photocatalytic activity. The D_PEF_ of g-C_3_N_4_/TCNQ-10 is 84.8%, the D_PEF_ of g-C_3_N_4_/TCNQ-20 is 91.6%, and the D_PEF_ of g-C_3_N_4_/TCNQ-30 is 73.4% after doping different qualities of TCNQ. The results showed that adding TCNQ could effectively inhibit the recombination of h^+^ and e^−^, enhancing the photocatalytic activity of g-C_3_N_4_/TCNQ. In the g-C_3_N_4_/TCNQ-20 sample, the degradation rate of PEF can reach 91.6%, and the catalytic activity is the best.

### 3.11. Influence of Catalyst Dosage on Catalytic Effect of PEF

**Figure 11:**
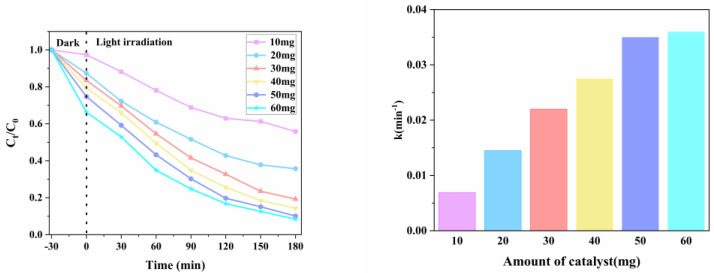
Influence of catalyst usage on the catalytic effect of PEF.

Figure 11 shows the photocatalytic effect diagram of samples with different masses of g-C_3_N_4_/TCNQ-20 at a PEF of 10 mg/L. 10 mg D_PEF_ = 44.16%, 20 mg D_PEF_ = 64.31%, 30 mg D_PEF_ = 80.69%, 40 mg D_PEF_ = 85.73%, 50 mg D_PEF_ = 90.07%, 60 mg D_PEF_ = 91.32%. As can be seen from the figure, the best addition was 60 mg and the worst addition was 10 mg as the amount of g-C_3_N_4_/TCNQ-20 samples added decreased. This change is because the more significant the amount of g-C_3_N_4_/TCNQ-20 that is used under the same illumination, the more photogenerated electron holes are generated. That is, more active substances are generated. Therefore, with the increase in the dosage, its degradation effect will also be enhanced. However, when the amount of catalyst is large, the increased value of the catalytic reaction speed is low. When the amount of catalyst is increased from 50 mg to 60 mg, the catalytic reaction speed is not significantly improved. Because the amount of g-C_3_N_4_/TCNQ-20 is too large, it will obviously scatter and reflect visible light, reduce the absorption of visible light, and then reduce the utilization rate of visible light, resulting in a decrease in the ability of photocatalytic degradation. Therefore, 50 mg of g-C_3_N_4_/TCNQ-20 is the optimal dosage.

### 3.12. Kinetic Evaluation of the Photocatalytic Process of Catalyst PEF Degradation

**Figure 12:**
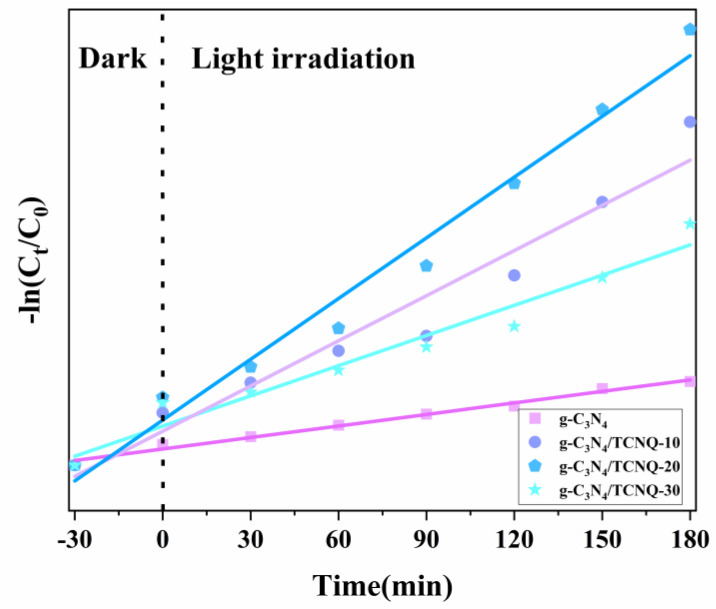
First-order reaction constants of g−C_3_N_4_/TCNQ−mediated degradation of PEF.

Figure 12 shows the photocatalytic degradation kinetics of PEF, which was fitted using the first-order kinetic method. The composite sample was exposed to a dark reaction in PEF for 30 min to reach the adsorption–desorption equilibrium. The end time of the reaction in the dark and the start time of the reaction in the light was set to zero, and the kinetics of the photocatalytic reaction were studied. The conclusion is that the relationship between ln(C_0_/C_t_) and the photocatalytic reaction time t is linear for each sample.

As shown in Table 1, the k value of g-C_3_N_4_/TCNQ-20 is greater, k = 0.0111 min^−1^, and has the highest degree of PEF degradation, which is about four times that of g-C_3_N_4_. The R^2^ of each g-C_3_N_4_/TCNQ composite sample is greater than 0.9, thus concluding that each sample satisfies the match between the D_PEF_ and the first-order reaction kinetic equation.

### 3.13. Analysis of Experimental Results for Reuse of PEF Degradation

**Figure 13:**
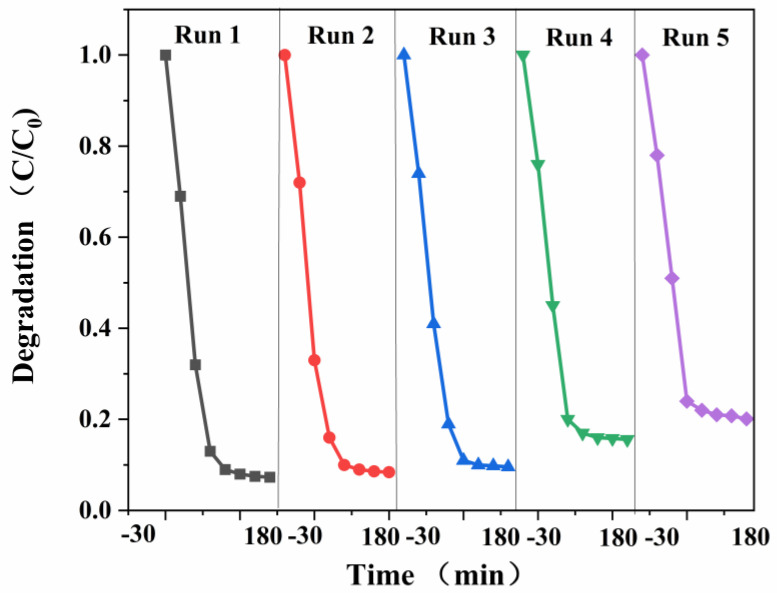
Reuse experiment of g−C_3_N_4_/TCNQ composite material degrading PEF.

**Figure 14:**
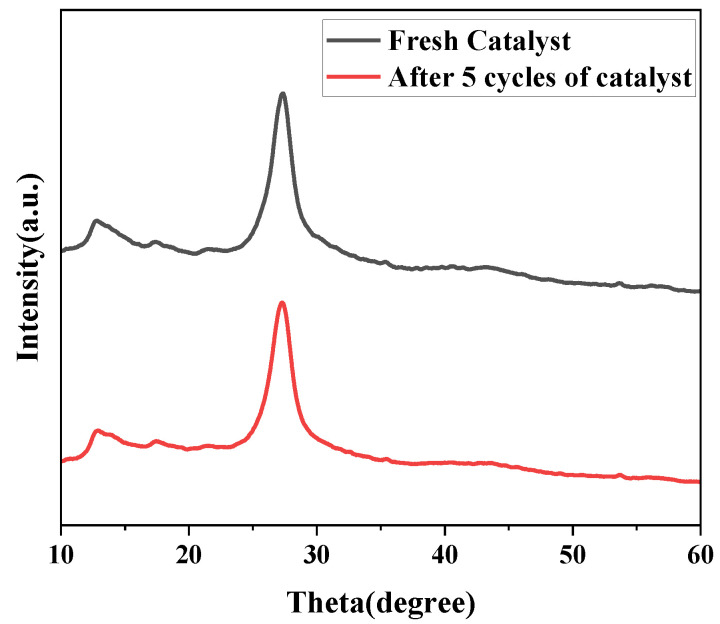
XRD patterns of g-C_3_N_4_/TCNQ samples before and after reuse.

Figure 13 shows the catalyst stable cycle diagram, where the used g-C_3_N_4_/TCNQ-20 photocatalyst was collected by centrifugation and washed three times with water and alcohol. After drying in an oven at 60 °C, an equal amount of dry powder was used as a photocatalyst for the next cycle run. After repeating the experiment four times, the first degradation effect was the best, and then the degradation rate each time decreased. However, the change was insignificant, and the catalyst still had a high degradation rate of about 80%. The results show that the catalyst has good stability, renewable performance, and can be reused. Furthermore, the XRD spectra of the reused catalysts (Figure 14) showed no significant changes in their crystal properties, which indicated the comparative stability of the prepared catalysts. The results indicate that g-C_3_N_4_/TCNQ is an efficient and stable visible-light-driven photocatalyst for the degradation of quinolones.

### 3.14. Analysis of Free Radical Capture Experimental Results of PEF Degradation by Catalyst

**Figure 15:**
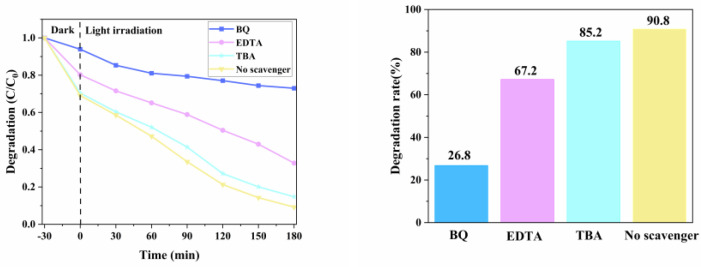
Effect of different radical capture agents on catalytic degradation.

Free radical trapping experiments can analyze the main active components in photocatalysis, and the possible mechanism is discussed. In this study, three different trapping agents, 1,4-benzoquinone (BQ), tert-butyl alcohol (TBA), ethylenediamine tetraacetate sodium (EDTA-2Na), were used to capture superoxide radical (·O^2−^), hydroxyl radical (·OH), and hole (h^+^), respectively. The experimental results are shown in Figure 15. It can be seen from the figure that when no capture agent is added, the degradation rate of PEF by g-C_3_N_4_/TCNQ-20 can still reach 90.8%. When the capture agents BQ, TBA, and EDTA are added, the degradation rate drops to 26.8%, 85.2%, and 67.2%, respectively. When BQ was added to the system to remove superoxide radicals, the photocatalytic degradation rate was significantly inhibited, indicating that superoxide radicals were the main active substances. At the same time, adding EDTA can also reduce the speed of the photocatalytic reaction, indicating that h^+^ also plays a vital role in the photocatalytic reaction. After the addition of TBA, the rate of its catalytic degradation did not decrease significantly compared with that without the addition of the capture agent, which indicated that the elimination of ·OH did not significantly influence the photocatalytic reaction. Therefore, ·O^2−^ and h^+^ are the main active species in the g-C_3_N_4_/TCNQ catalytic system. Among them, ·O^2−^ is more critical for PEF degradation.

### 3.15. Photocatalytic Mechanism Analysis

**Figure 16:**
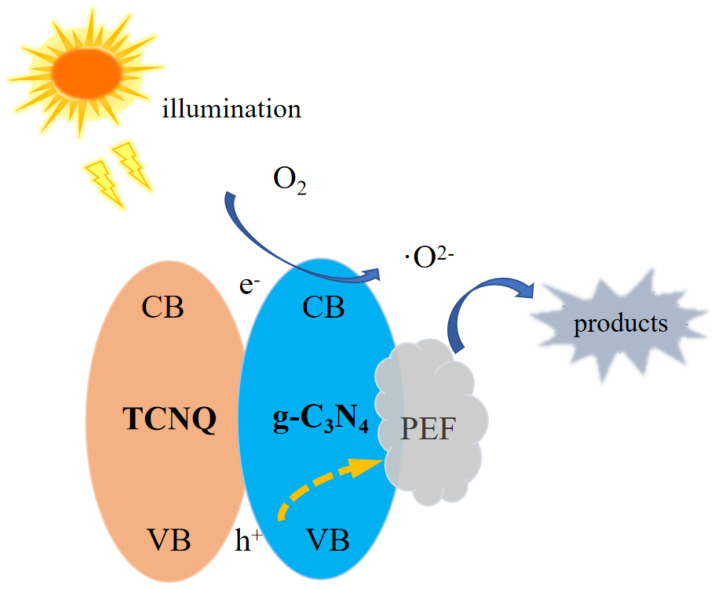
Possible degradation mechanism of PEF.

It can be seen from the above results that the photocatalytic activity of the g-C_3_N_4_/TCNQ composite sample is higher than that of the g-C_3_N_4_. On this basis, it is hypothesized how the g-C_3_N_4_/TCNQ composite sample photo-catalytically degrades PEF. Since the g-C_3_N_4_/TCNQ sample with a lower gap width than g-C_3_N_4_ is more likely to accept lower photon energy intensity, the electrons on VB (e^−^) jump into CB through the gap and form a photogenerated carrier hole (e^−^-h^+^). The photogenerated electrons on CB are transferred to VB on the material’s surface, e^−^ reacts with O_2_ in solution to generate·O^2−^, and·O^2−^ reacts with PEF to generate intermediate substances and then generates small molecular substances. On the other hand, h^+^ on VB directly reacts with PEF, and then catalyzes PEF degradation to increase the composite sample’s capacity for photocatalysis. Figure 16 shows the possible mechanism for PEF degradation.

## 4. Conclusions

In this paper, a g-C_3_N_4_, g-C_3_N_4_/TCNQ complex was successfully prepared using the coprothermal method, and characterized by XRD, FT-IR, SEM, TEM, DRS, PL, and I-T. PEF was employed as a degradation product to assess the catalyst’s photocatalytic properties, and PEF was broken down under visible light (>550 nm) to examine the photocatalytic activity. When the doping amount of g-C_3_N_4_/TCNQ-20 was 50 mg, the D_PEF_ was 91.6%. All of the sample pairs of D_PEF_ conformed to the first-order kinetic equation when combined with the kinetic analysis of the photocatalytic reaction, four times more than g-C_3_N_4_ alone. After five cycles, the photodegradation activity of the g-C_3_N_4_/TCNQ composite sample was still as high as 80%. The free radical capture experiments found that ·O^2−^ was the main active species of the g-C_3_N_4_/TCNQ catalytic system, h^+^ also plays a role in the degradation of PEF. This study provides an advanced and green method for the degradation of antibiotics in wastewater and provides a theoretical basis for improving the pollution of antibiotic wastewater.

## Figures and Tables

**Table 1:** Linear fitting data of photocatalytic degradation kinetics of PEF in g-C_3_N_4_/TCNQ composite samples.

Sample Name	Regression Equation	*k*	*R* ^2^
g-C_3_N_4_	*y* = 0.0021*x* + 0.0885	0.0021	0.9888
g-C_3_N_4_/TCNQ-10	*y* = 0.0083*x* + 0.1862	0.0083	0.9523
g-C_3_N_4_/TCNQ-20	*y* = 0.0111*x* + 0.2471	0.0111	0.9800
g-C_3_N_4_/TCNQ-30	*y* = 0.0055*x* + 0.2143	0.0055	0.9578

## Data Availability

The data is unavailable due to privacy or ethical restrictions.
